# Serum neuregulin 4 is negatively correlated with insulin sensitivity in humans and impairs mitochondrial respiration in HepG2 cells

**DOI:** 10.3389/fphys.2022.950791

**Published:** 2022-09-15

**Authors:** Cristina Martínez, Jèssica Latorre, Francisco Ortega, María Arnoriaga-Rodríguez, Aina Lluch, Núria Oliveras-Cañellas, Francisco Díaz-Sáez, Julian Aragonés, Marta Camps, Anna Gumà, Wifredo Ricart, José Manuel Fernández-Real, José María Moreno-Navarrete

**Affiliations:** ^1^ Department of Diabetes, Endocrinology and Nutrition, Institut d’Investigació Biomèdica de Girona, Girona, Spain; ^2^ CIBEROBN (CB06/03/010), Instituto de Salud Carlos III, Madrid, Spain; ^3^ Departament de Bioquímica i Biomedicina Molecular, Facultat de Biologia, Universitat de Barcelona (UB), Barcelona, Spain; ^4^ Institut de Biomedicina de la Universitat de Barcelona (IBUB), Universitat de Barcelona (UB), Barcelona, Spain; ^5^ Research Unit, Hospital of Santa Cristina, Research Institute Princesa, Autonomous University of Madrid, Madrid, Spain; ^6^ CIBER de Enfermedades Cardiovasculares, Carlos III Health Institute, Madrid, Spain; ^7^ CIBER de Diabetes y Enfermedades Metabólicas Asociadas, Instituto de Salud Carlos III, Madrid, Spain; ^8^ Department of Medicine, University of Girona, Girona, Spain

**Keywords:** neuregulin 4, obesity, insulin resistance, mitochondrial respiration, HepG2 cells

## Abstract

Neuregulin 4 (NRG4) has been described to improve metabolic disturbances linked to obesity status in rodent models. The findings in humans are controversial. We aimed to investigate circulating NRG4 in association with insulin action in humans and the possible mechanisms involved. Insulin sensitivity (euglycemic hyperinsulinemic clamp) and serum NRG4 concentration (ELISA) were analysed in subjects with a wide range of adiposity (*n* = 89). *In vitro* experiments with human HepG2 cell line were also performed. Serum NRG4 was negatively correlated with insulin sensitivity (*r* = −0.25, *p* = 0.02) and positively with the inflammatory marker high-sensitivity C reative protein (hsCRP). In fact, multivariant linear regression analyses showed that insulin sensitivity contributed to BMI-, age-, sex-, and hsCRP-adjusted 7.2% of the variance in serum NRG4 (*p* = 0.01). No significant associations were found with adiposity measures (BMI, waist circumference or fat mass), plasma lipids (HDL-, LDL-cholesterol, or fasting triglycerides) or markers of liver injury. Cultured hepatocyte HepG2 treated with human recombinant NRG4 had an impact on hepatocyte metabolism, leading to decreased gluconeogenic- and mitochondrial biogenesis-related gene expression, and reduced mitochondrial respiration, without effects on expression of lipid metabolism-related genes. Similar but more pronounced effects were found after neuregulin 1 administration. In conclusion, sustained higher serum levels of neuregulin-4, observed in insulin resistant patients may have deleterious effects on metabolic and mitochondrial function in hepatocytes. However, findings from *in vitro* experiments should be confirmed in human primary hepatocytes.

## Introduction

Neuregulins are members of the large epidermal growth factors (EGF) family of proteins, encrypted by four different genes (NRG1-4) that encode multiple isoforms characterized by the presence of an EGF-like domain that mediates their biological activity through binding to the receptors tyrosine kinase ErbB3 and ErbB4 (Meyer et al., 1997; Gumà et al., 2020). The role of neuregulins in energy balance, glucose and lipid metabolism, and their implication in metabolic syndrome, have been investigated in recent years. ErbB receptors have been demonstrated to be expressed in skeletal myocytes, with NRG1 being key to skeletal muscle development, myogenesis, and regulation of muscle metabolism by stimulating glucose utilization (Suárez et al., 2001; Cantó et al., 2007; Caillaud et al., 2016; Ennequin et al., 2017; Heim et al., 2020), and in the proliferation of cardiomyocytes during heart regeneration (Honkoop et al., 2019). Recombinant neuregulin administration in Zucker diabetic fatty rats enhanced glucose tolerance through the activation of the ErbB3/PI3K/PKB signalling pathway in liver, but not in muscle (López-Soldado et al., 2016). In addition, a recent study demonstrated that NRG4 is required for insulin-induced glucose uptake in mouse 3T3-L1 cells (Díaz-Sáez et al., 2021). NRG4 or ErbB4-induced downregulation led to insulin resistance and hepatic steatosis in high-fat diet-fed mice (Wang et al., 2014; Zeng et al., 2018; Wang et al., 2019a; Zhu et al., 2020), supporting the importance of NRG4-ErbB4 signalling in the prevention of obesity-associated metabolic disturbances.

In humans, the association between NRG4 and obesity-associated insulin resistance and liver steatosis is less clear and controversial. Some studies demonstrated an inverse association between circulating NRG4 concentration and characteristics of the metabolic syndrome or the presence of non-alcoholic fatty liver disease (Dai et al., 2015; Wang et al., 2019b). Other investigations reported increased levels of NRG4 in patients with type 2 diabetes, altered glucose tolerance or obesity (Kang et al., 2016; Chen et al., 2017a; Kurek Eken et al., 2018). Chen et al. (2017a) analysed serum NRG4 in 310 subjects (83 with normal glucose tolerance, 129 with prediabetes and 96 with type 2 diabetes). Circulating NRG4 was significantly increased in the prediabetic and diabetic groups. Kurek Eken et al. (2018) also described increased serum NRG4 levels in women with gestational diabetes mellitus in association with increased BMI, glucose at 2-h of an oral glucose tolertance test and HOMA-IR. Kang et al. (2016) also found increased serum levels of NRG4 in participants with overweight (mean BMI 27 ± 4.02 kg/m^2^) and type 2 diabetes compared to participants without obesity (mean BMI 24.1 ± 2.65 kg/m^2^). Consistently, a recent meta-analysis, in which seven studies were included, concluded that circulating NRG4 was associated with alterations in glucose metabolism and obesity (Wang et al., 2019c).

No study has evaluated, to our knowledge, serum NRG4 in association with gold standard measures of insulin action (euglycemic hyperinsulinemic clamp) or its possible mechanisms in human cells. In the present study, we aimed to investigate the potential relationship between circulating NRG4 and obesity-associated metabolic disturbances in non-diabetic subjects with a wide range of adiposity. We also evaluated the metabolic impact of neuregulins and the effects of NRG1 and NRG4 on palmitate-treated hepatocytes of the human HepG2 cell line.

## Methods

### Participants recruitment

From January 2016 to October 2017, a cross-sectional case-control study was undertaken in the Endocrinology Department of Josep Trueta University Hospital. We included 89 consecutive subjects, 55 with obesity (BMI≥30 kg/m^2^) participants, and 34 without obesity (BMI<30 kg/m^2^) similar in age (age range of 28–66 years) and sex distribution. Exclusion criteria were type 2 diabetes, clinically significant hepatic, neurological, or other major systemic disease, including malignancy, infection in the previous month, an elevated serum creatinine concentration, acute major cardiovascular event in the previous 6 months, acute illnesses and current evidence of high grade chronic inflammatory or infective diseases, serious chronic illness, >20 g ethanol intake/day, or use of medications that might interfere with insulin action. Liver and thyroid dysfunction were specifically excluded by biochemical work-up. Samples and data from patients included in this study were provided by the FATBANK platform promoted by the CIBEROBN and coordinated by the IDIBGI Biobank (Biobanc IDIBGI, B.0000872), integrated in the Spanish National Biobanks Network and they were processed following standard operating procedures with the appropriate approval of the Ethics, External Scientific and FATBANK Internal Scientific Committees. To ensure blinding in outcome analyses, all samples were codified.

The institutional review board—Ethics Committee and the Committee for Clinical Research (CEIC) of Dr. Josep Trueta University Hospital (Girona, Spain) approved the study protocol and informed written consent was obtained from all participants.

### Anthropometric measurements and analytical methods

BMI was calculated as the weight in kilograms divided by height in meters squared. The waists of participants were measured with a soft tape midway between the lowest rib and the iliac crest, and hip circumference was measured at the widest part of the gluteal region. Body composition was assessed using a dual energy X-ray absorptiometry (DEXA, GE lunar, Madison, Wisconsin). Serum glucose concentrations were measured in duplicate by the glucose oxidase method using a Beckman glucose analyzer II (Beckman Instruments, Brea, California). Glycated haemoglobin (HbA1c) was measured by the high-performance liquid chromatography method (autoanalyser Jokoh HS-10, Bio-Rad, Muenchen, Germany). HDL-cholesterol was quantified following precipitation with polyethylene glycol at room temperature. The Friedewald formula was used to calculate the concentration of LDL-cholesterol. Total serum triglycerides were measured through the reaction of glycerol-phosphate-oxidase and peroxidase on a Hitachi 917 instrument (Roche, Mannheim, Germany). High-sensitivity (hs) C-reactive protein (ultrasensitive assay; 110 Beckman, Fullerton, CA), alanine aminotransferase (ALT), aspartate aminotransferase (ASP) and gamma glutamyl transferase (GGT) were determined by a routine laboratory test. Serum neuregulin 4 (NRG4) concentrations were measured using and enzyme-linked immunosorbent assay (ELISA) kits (Aviscera Bioscences, Santa Clara, CA). This assay has been shown to be highly sensitive to human NRG4 with a sensitivity of 0.25 ng/ml. Intra- and inter-assay variations were both less than 10%.

### Hyperinsulinemic-euglycemic clamp

Insulin action was determined using the hyperinsulinemic-euglycemic clamp. After an overnight fast, two catheters were inserted into an antecubital vein, one for each arm, used to administer constant infusions of glucose and insulin and to obtain arterialized venous blood samples. A 2-h hyperinsulinemic-euglycemic clamp was initiated by a two-step primed infusion of insulin (80 mU/m^2^/min for 5 min, 60 mU/m^2^/min for 5 min) immediately followed by a continuous infusion of insulin at a rate of 40 mU/m^2^/min (regular insulin [Actrapid; Novo Nordisk, Plainsboro, NJ]). Glucose infusion began at minute four at an initial perfusion rate of 2 mg/kg/min being then adjusted to maintain plasma glucose concentration at 88.3–99.1 mg/dl. Blood samples were collected every 5 min for determination of plasma glucose and insulin. Insulin sensitivity was assessed as the mean glucose infusion rate during the last 40 min. In the stationary equilibrium, the amount of glucose administered (M) equals the glucose taken by the body tissues and is a measure of overall insulin sensitivity.

### *In vitro* experiments

Human hepatoma HepG2 cells were purchased from American Type Culture Collection (ATCC) and cultured in Dulbecco’s Modified Eagle’s Medium (DMEM) supplemented with 10% fetal bovine serum (Gibco), 100 units ml–1 penicillin and streptomycin, 1% glutamine and 1% sodium pyruvate, at 37°C and 5% CO_2_ atmosphere. Palmitic acid (PA) was prepared as follows: 27.84 mg of PA (Sigma, San Luis, MO) were dissolved in 1 ml sterile water to make a 100 mM stock solution. Bovine serum albumin (BSA, 5%) was prepared in serum-free DMEM. 100 mM PA stock solution and 5% BSA were mixed for at least 1 h at 40°C to obtain a 5 mM solution. Cells were treated with PA 500 uM in combination with each of the neuregulin, human recombinant NRG4 (Cat nº RKQ8WWG1, Reprokine Ltd., Rehobot, Israel) and NRG1 (Cat nº 396-HB, R&D Systems, Inc., MN, United States) at 50 ng/ml during 48 h. The functionality of the dose and incubation period of these proteins was based on the validation performed in a previous study (Díaz-Sáez et al., 2021). BSA supplemented medium was used as control when necessary. All experimental conditions were performed in four biological replicates in two independent experiments. After treatment, cells were washed with phosphate buffered saline and collected with Qiazol for RNA purification.

### Gene expression analysis

RNA purification, gene expression procedures and analyses were performed, as previously described (Ortega et al., 2015). Briefly, RNA purification was performed using RNeasy Lipid Tissue Mini Kit (QIAgen, Izasa SA, Barcelona, Spain) and the integrity was checked by Agilent Bioanalyzer (Agilent Technologies, Palo Alto, CA). Gene expression was assessed by real time PCR using a LightCycler^®^ 480 Real-Time PCR System (Roche Diagnostics SL, Barcelona, Spain), using TaqMan*®* and SYBR green technology suitable for relative genetic expression quantification. The RT-PCR reaction was performed in a final volume of 12 μl. The cycle program consisted of an initial denaturing of 10 min at 95°C then 40 cycles of 15 s denaturizing phase at 95°C and 1 min annealing and extension phase at 60°C. A threshold cycle (Ct value) was obtained for each amplification curve and then a ΔCt was first calculated by subtracting the Ct value for human cyclophilin A (*PPIA*) RNA from the Ct value for each sample. Fold changes compared with the endogenous control were then determined by calculating 2^−ΔCt^, so that gene expression results are expressed as expression ratio relative to *PPIA* gene expression according to the manufacturer’s guidelines. TaqMan^®^ primer/probe sets (Thermo Fisher Scientific, Waltham, MA, United States) used were as follows: Peptidylprolyl isomerase A (cyclophilin A) (4333763, *PPIA* as endogenous control), phosphoenol pyruvate carboxykinase 1 (*PEPCK* or *PCK1*, Hs0159918_m1), glucose-6-phosphatase (*G6PC*, Hs00609178_m1), glucose transporter 2 or solute carrier family two member 2 (*GLUT2* or *SLC2A2*, Hs01096908_m1). CD36 molecule (*CD36*, Hs00169627_m1), acyl-CoA synthetase long chain family member 1 (*ACSL1*, Hs00960561_m1), stearoyl-Coenzyme A desaturase 1 (*SCD1*, Hs01682761_m1), PPAR*g* coactivator one alpha (*PPARGC1A*, Hs00173304_m1), nuclear respiratory factor 1 (*NRF*1, Hs00192316_m1), glucose transporter 4 or solute carrier family two member 4 (*GLUT*4 or *SLC2A4*, Hs00168966_m1) and fatty acid synthase (*FASN*, Hs01005622_m1).

### Mitochondrial respiration

Mitochondrial respiratory function was assessed in HepG2 treated with PA in combination with either NRG1 or NRG4 by means of a Seahorse XFp Extracellular Flux Analyzer (Seahorse Bioscience, Agilent Technologies) using SeahorseXFp Cell MitoStress Test Kit according to manufacturer’s instructions. This assay determines basal respiration, ATP production, H+ (proton) leak, and spare respiratory capacity. Basal respiration shows energy demand of the cell under baseline conditions. ATP production shows ATP synthesized by the mitochondria. Proton leak is the remaining basal respiration not coupled to ATP production and can be a sign of mitochondrial damage. Maximal respiration shows the maximum rate of respiration that the cell can achieve. Spare respiratory capacity indicates the capability of the cell to respond to an energy demand and can be an indicator of cell fitness or flexibility. Cells were cultured for 48 h, followed by 60 min of culture with XF base medium supplemented with 1 mM pyruvate, 2 mM glutamine, and 10 mM glucose in a CO_2_ free incubator. Oxygen consumption rate (OCR) was then normalized to the total protein content, determined by PierceTM BCA Protein Assay Kit (Thermo Fisher Scientific, Wilmington, DE).

### Statistical analyses

Statistical analyses were performed using SPSS 12.0 software. Unless otherwise stated, descriptive results of continuous variables are expressed as mean and SD for Gaussian variables or median and interquartile range. Normality analysis was conducted using the Kolmogorov-Smirnov test. Unpaired *t*-test was used to compare serum NRG4 concentration according to obesity. The correlation between variables was analyzed using simple correlation analyses (Pearson’s and Spearman’s test) and multiple regression analysis. *In vitro* experiments were analysed using non-parametric Mann-Whitney test. Levels of statistical significance were set at *p* < 0.05.

## Results

### Serum NRG4 associations with metabolic traits and insulin resistance

**FIGURE 1:**
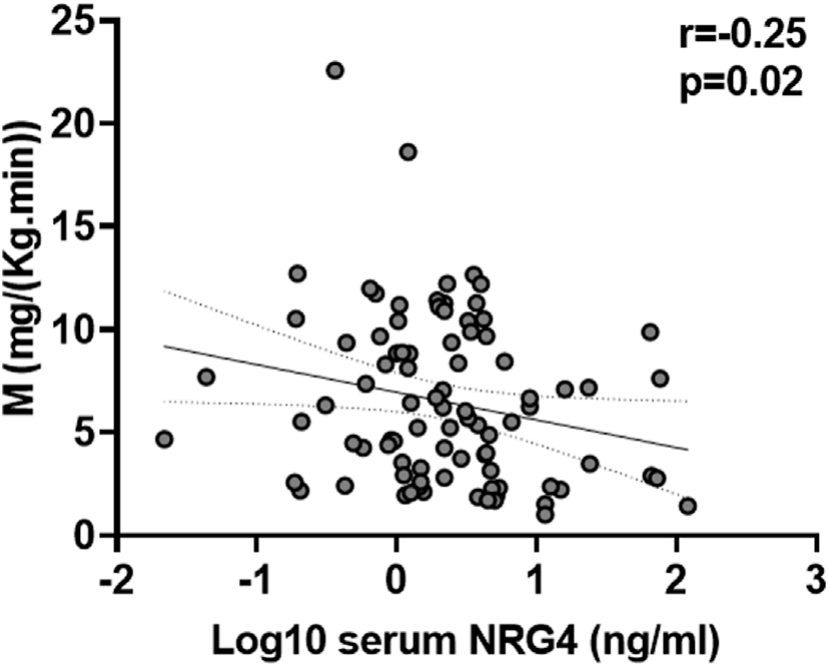
Bivariate correlation between serum NRG4 levels and insulin sensitivity in all participants (*N* = 89).

Serum NRG4 was negatively correlated with insulin sensitivity (M) (Figure 1) and positively with the inflammatory marker hsCRP (Table 1). Multivariant linear regression analyses showed that insulin sensitivity contributed to 7.2% of the variance in serum NRG4 after controlling for BMI, age, sex and hsCRP (*p* = 0.01) (Table 2).

**TABLE 1:** Anthropometric and clinical characteristics according to obesity and correlations between serum NRG4 and these parameters.

	Non obese	Obese	*p*	r	p*
N (men/women)	9/25	16/39			
Age (years)	49.0 ± 10.3	44.4 ± 10.6	**0.04**	−0.10	0.32
Body mass index (kg/m^2^)	24.6 ± 2.7	44.2 ± 7.3	**<0.0001**	0.15	0.13
Waist circumference (cm)	88.7 ± 9.4	126.9 ± 14.3	**<0.0001**	0.11	0.30
BI fat mass (%)	25.2 ± 6.0	44.0 ± 5.0	**<0.0001**	0.14	0.17
Cholesterol (mg/dl)	209.2 ± 48.4	195.5 ± 44.0	0.17	0.01	0.87
HDL (mg/dl)	69.6 ± 22.0	50.2 ± 12.8	**<0.0001**	−0.13	0.19
LDL (mg/dl)	126.4 ± 39.6	122.4 ± 35.6	0.62	0.06	0.52
Triglycerides (mg/dl)^a^	93.8 (60.7–107)	116.1 (69–142)	**0.05**	0.16	0.11
ASP (U/L)	22.0 ± 6.0	20.2 ± 7.1	0.24	0.13	0.20
ALT (U/L)	21.3 ± 9.1	25.3 ± 14.7	0.15	0.09	0.38
GGT (U/L)^a^	32.2 (11.5–32.5)	28.7 (18–32)	0.62	0.19	0.06
Glucose (mg/dl)	95.6 ± 12.3	95.6 ± 11.6	0.98	0.09	0.37
Glycated haemoglobin (%)	5.4 ± 0.2	5.5 ± 0.3	0.19	0.16	0.11
M [mg/(kg min)]	9.8 ± 3.8	4.4 ± 2.5	**<0.0001**	−0.25	**0.02**
hsCRP (mg/dl)^a^	1.4 (0.51–1.74)	8.2 (2.6–10.5)	**<0.0001**	0.21	**0.04**
NRG4 (ng/ml)^a^	2.33 (0.79–3.6)	3.4 (0.95–4.5)	0.13	—	—

a Median and interquartile range. Bold values mean that *p*-value reached statistical significance. p* indicated the p-value of correlations.

**TABLE 2:** Multiple linear regression analysis to predict the impact of insulin sensitivity on serum NRG4 after controlling for BMI, age, sex, and hsCRP.

	NRG4 (ng/ml)		
	*β*	t	*p*
Age (years)	−0.070	−0.63	0.5
Gender	0.013	0.11	0.9
BMI (kg/m^2^)	0.044	0.26	0.8
hsCRP (mg/dl)	0.066	0.54	0.6
M [mg/(kg min)]	−0.289	−2.65	**0.01**
Adjusted *R* ^2^	0.072 (7.2%)		
ANOVA *P*	**0.01**		

Bold values mean that *p*-value reached statistical significance.

No significant associations were observed between serum NRG4 levels and adiposity measures (BMI, waist circumference or fat mass), plasma lipids (HDL, LDL-cholesterol, and fasting triglycerides), or markers of liver injuty (AST, ALT, GGT) (Table 1).

### Human recombinant NRG4 and NRG1 reduce expression of gluconeogenic- and mitochondrial biogenesis-related genes in palmitate-treated HepG2 cells

**FIGURE 2:**
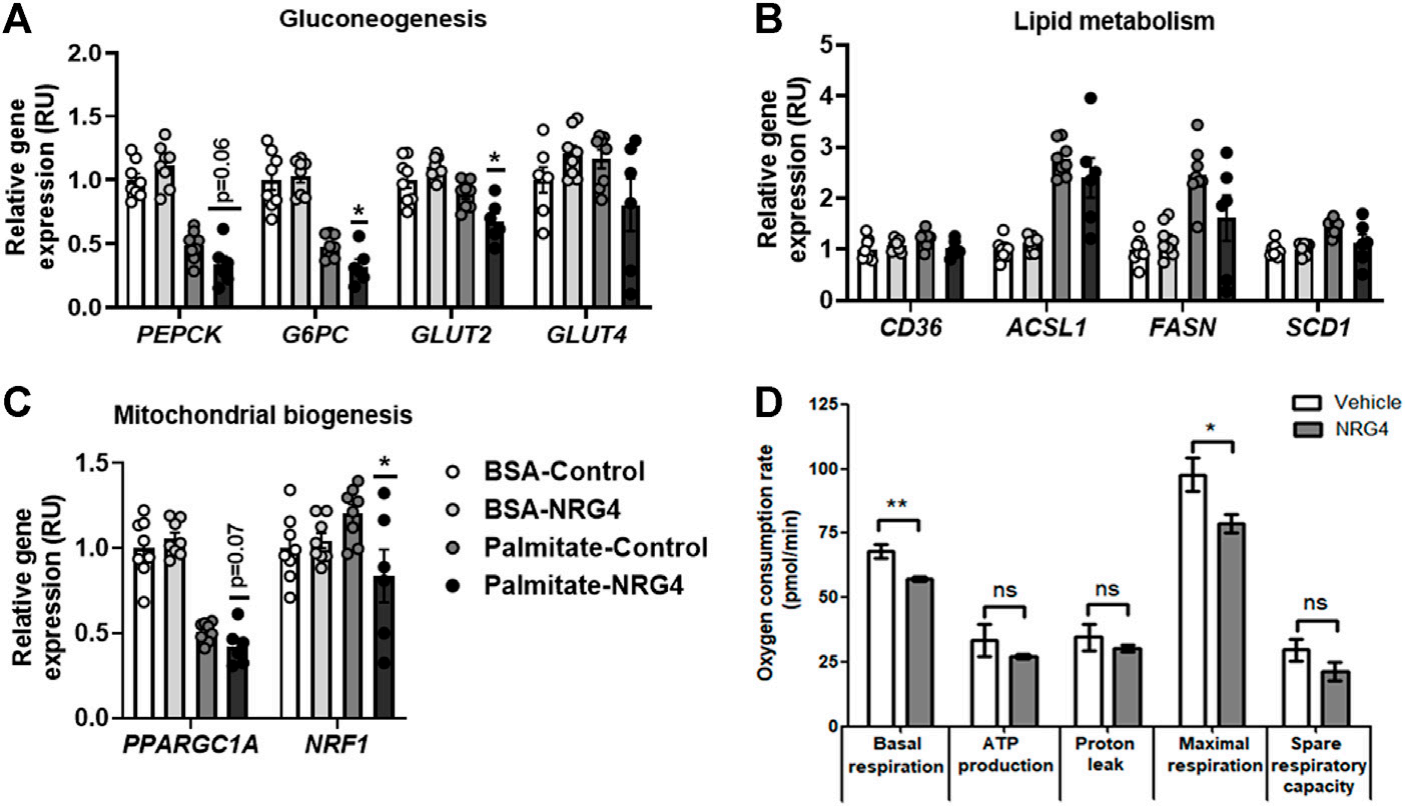
**(A–C)** The impact of human recombinant NRG4 on gluconeogenesis-, lipid metabolism- and mitochondrial biogenesis-related gene expression in BSA- and palmitate-treated HepG2 cells. **(D)** The impact of human recombinant NRG4 on mitochondrial respiration in palmitate-treated HepG2 cells. *p < 0.05 and **p < 0.01 compared to control or vehicle.

**FIGURE 3:**
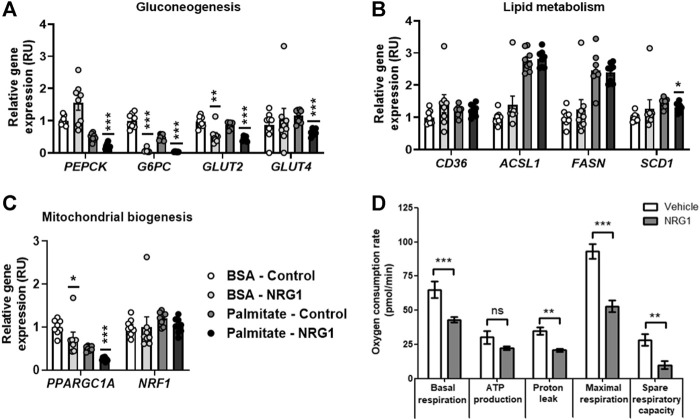
**(A–C)** The impact of human recombinant NRG1 on gluconeogenesis-, lipid metabolism-, and mitochondrial biogenesis-related gene expression in BSA- and palmitate-treated HepG2 cells. **(D)** The impact of human recombinant NRG1 on mitochondrial respiration in palmitate-treated HepG2 cells. *p < 0.05, **p < 0.01, **p < 0.001 compared to control or vehicle.

Recombinant NRG1 administration was performed to test if the putative effects of NRG4 on HepG2 were specific or similar to other neuregulins. Both human recombinant NRG4 or NRG1 administration resulted in decreased gluconeogenic- and mitochondrial biogenesis-related gene expression, being more pronounced these effects after NRG1 administration in palmitate-treated HepG2 cells (Figures 2, 3).

After vehicle (BSA), no significant effects of human recombinant NRG4 on gluconeogenic (*PEPCK, G6PC, GLUT2, GLUT4*)-, lípid metabolism (*CD36, ACSL1, FASN, SCD1*)- and mitochondrial biogenesis (*PPARGC1A, NRF1*)-related gene expression were found (Figure 2). Human recombinant NRG1 led to decreased *G6PC, GLUT2* and *PPARGC1A* mRNA levels (Figure 3).

### Human recombinant NRG4 and NRG1 attenuate mitochondrial respiration in palmitate-treated HepG2 cells

After 24 h-palmitate administration, NRG4 administration led to reduced basal and maximal respiration, without significant effects on oxygen consumption for ATP production and proton leak and on spare respiratory capacity (Figure 2). NRG1 administration led to reduced basal and maximal respiration, proton leak and spare respiratory capacity (Figure 3).

## Discussion

In the current study we found that serum NRG4 was positively associated with insulin resistance and hsCRP (a marker of chronic low-level inflammation), but not with dyslipidemia or markers of liver injury in subjects without type 2 diabetes. Importantly, the relationship of NRG4 with insulin sensitivity remained significant after controlling for age, BMI, sex and hsCRP. In line with the current study, increased levels of serum NRG4 in insulin resistance-associated diseases, such as type 2 diabetes (Kang et al., 2016; Chen et al., 2017a; Kurek Eken et al., 2018; Wang et al., 2019c; Kocak et al., 2019) and polycystic ovary syndrome (Kurek Eken et al., 2019; Cao and Hu, 2021) have been reported. In contrast, a recent study described low levels of NRG4 in patients with type 2 diabetes in association to microalbuminuria (Kocak et al., 2020), suggesting NRG4 as a putative marker of microvascular dysfunction. Increased levels of NRG4 in situations of altered glucose tolerance or early diabetes (Kang et al., 2016; Chen et al., 2017a; Kurek Eken et al., 2018; Wang et al., 2019c; Kocak et al., 2019), but decreased in those patients with advanced diabetes (as reflects increased microalbuminuria) (Kocak et al., 2020), suggest that NRG4 levels might depend on the evolution and stage of development of diabetes. Taking into account the importance of NRG4 in insulin-induced glucose uptake (Díaz-Sáez et al., 2021), a possible compensatory role of NRG4 in the maintenance of insulin action and glucose metabolism in situations of early diabetes is possible. However, additional interventional studies should be performed to clarify the relevance of increased serum NRG4 in early diabetes.

Controversial findings from previous studies (Dai et al., 2015; Kang et al., 2016; Chen et al., 2017a; Kurek Eken et al., 2018) together with current observations suggest that NRG4 does not exert in humans the benefitial effects previously demonstrated in mice (Wang et al., 2014; Chen et al., 2017b). To further evaluate the possible mechanisms involved, *in vitro* experiments in the HepG2 cell line were performed. These experiments revealed that, after 24 h-palmitate administration, human recombinant NRG4 did not alter lipid metabolism-related gene expression. In contrast to previous findings in mice (Wang et al., 2014; Chen et al., 2017b; Wang et al., 2019a), NRG4 impacted negatively on hepatocyte catabolism, attenuating the expression of gluconeogenesis- and mitochondrial biogenesis-related genes and slightly decreasing mitochondrial respiration. Of note, these effects were even more pronounced after human recombinant NRG1 administration, indicating that both NRG4 and NRG1 impacts negatively on gluconeogenesis and mitochondrial respiration in human hepatocytes exposed to palmitate. In line with these findings, previous studies in mice demonstrated that recombinant NRG1 administration attenuated hepatic gluconeogenesis (Arai et al., 2017; Zhang et al., 2018). The negative effects of NRG1 and NRG4 on mitochondrial biogenesis and respiration and the absence of impact on expression of lipid metabolism-related genes suggest that neuregulins did not prevent lipid accumulation in human hepatocytes as observed in mice (Wang et al., 2014; Chen et al., 2017b; Wang et al., 2019a; Zhu et al., 2020; Yang et al., 2021). The lack of protein-based data is a limitation of these experiments. While gene expression analysis at mRNA level is a suitable surrogate to characterize the effect of exogenous factors on gluconeogenesis, lipogenesis and other metabolic pathways in HepG2 cells (Patel et al., 2016; Hasei et al., 2021; Wang et al., 2022), these data should be validated at protein level in additional experiments. Even though, HepG2 cells due to their proliferative capacity are very useful for exploratory *in vitro* experiments (Patel et al., 2016; Hasei et al., 2021; Wang et al., 2022), is important to note that compared to primary hepatocytes, these cells display altered expression and activation of EGFR and enhanced glycolytic pathway. To overcome these experimental limitations and confirm current findings, further experiments in human primary hepatocytes should be required.

The benefitial impact of NRG4 on metabolism observed in mice might be explained by the direct effects of NRG4 on adipose tissue, increasing sympathetic innervations, and the enhancement of BAT activity and browning of WAT (Rosell et al., 2014; Comas et al., 2019), but not on liver metabolism. In fact, while in mice the activation of BAT and the promotion of WAT browning ameliorate insulin sensitivity, liver steatosis and glucose tolerance in obesogenic conditions (Wang et al., 2015; Kimura et al., 2021), the impact of WAT browning on obesity or insulin resistance in humans is less relevant (Barquissau et al., 2018; Comas et al., 2019).

To sum up, NRG4 is negatively associated with insulin sensitivity in humans and contributes to reduce mitochondrial respiration in HepG2 hepatocytes.

## Data Availability

The original contributions presented in the study are included in the article/supplementary material, further inquiries can be directed to the corresponding authors.

## References

[B1] Arai T. Ono Y. Arimura Y. Sayama K. Suzuki T. Shinjo S. (2017). Type i neuregulin1α is a novel local mediator to suppress hepatic gluconeogenesis in mice. Sci. Rep. 7, 42959. 10.1038/srep42959 28218289 PMC5317163

[B2] Barquissau V. Léger B. Beuzelin D. Martins F. Amri E-Z. Pisani D. F. (2018). Caloric restriction and diet-induced weight loss do not induce browning of human subcutaneous white adipose tissue in women and men with obesity. Cell Rep. 22, 1079–1089. 10.1016/j.celrep.2017.12.102 29386128

[B3] Caillaud K. Boisseau N. Ennequin G. Chavanelle V. Etienne M. Li X. (2016). Neuregulin 1 improves glucose tolerance in adult and old rats. Diabetes Metab. 42, 96–104. 10.1016/j.diabet.2015.08.003 26404652

[B4] Cantó C. Pich S. Paz J. C. Sanches R. Martínez V. Orpinell M. (2007). Neuregulins increase mitochondrial oxidative capacity and insulin sensitivity in skeletal muscle cells. Diabetes 56, 2185–2193. 10.2337/db06-1726 17563068

[B5] Cao S. Hu Y. (2021). Effects of serum irisin, neuregulin 4, and weight management on obese adolescent girls with polycystic ovary syndrome. Biosci. Rep. 41, BSR20211658. 10.1042/BSR20211658 34427289 PMC8485390

[B6] Chen L. L. Peng M. M. Zhang J. Y. Hu X. Min J. Huang Q. L. (2017). Elevated circulating Neuregulin4 level in patients with diabetes. Diabetes. Metab. Res. Rev. 33, e2870. 10.1002/dmrr.2870 27862843

[B7] Chen Z. Wang G. X. Ma S. L. Jung D. Y. Ha H. Altamimi T. (2017). Nrg4 promotes fuel oxidation and a healthy adipokine profile to ameliorate diet-induced metabolic disorders. Mol. Metab. 6, 863–872. 10.1016/j.molmet.2017.03.016 28752050 PMC5518721

[B8] Comas F. Martínez C. Sabater M. Ortega F. Latorre J. Díaz-Sáez F. (2019). Neuregulin 4 is a novel marker of beige adipocyte precursor cells in human adipose tissue. Front. Physiol. 10, 39. 10.3389/fphys.2019.00039 30766490 PMC6365457

[B9] Dai Y. N. Zhu J. Z. Fang Z. Y. Zhao D. J. Wan X. Y. Zhu H. T. (2015). A case-control study: Association between serum neuregulin 4 level and non-alcoholic fatty liver disease. Metabolism. 64, 1667–1673. 10.1016/j.metabol.2015.08.013 26476959

[B10] Díaz-Sáez F. Blanco-Sinfreu C. Archilla-Ortega A. Sebastian D. Romero M. Hernández-Alvarez M. I. (2021). Neuregulin 4 downregulation induces insulin resistance in 3T3-L1 adipocytes through inflammation and autophagic degradation of GLUT4 vesicles. Int. J. Mol. Sci. 22, 12960. 10.3390/ijms222312960 34884763 PMC8657571

[B11] Ennequin G. Capel F. Caillaud K. Chavanelle V. Etienne M. Teixeira A. (2017). Neuregulin 1 improves complex 2-mediated mitochondrial respiration in skeletal muscle of healthy and diabetic mice. Sci. Rep. 7, 1742. 10.1038/s41598-017-02029-z 28496106 PMC5431817

[B12] Gumà A. Díaz-Sáez F. Camps M. Zorzano A. (2020). Neuregulin, an effector on mitochondria metabolism that preserves insulin sensitivity. Front. Physiol. 11, 696. 10.3389/fphys.2020.00696 32655416 PMC7324780

[B13] Hasei S. Yamamotoya T. Nakatsu Y. Ohata Y. Itoga S. Nonaka Y. (2021). Carnosic acid and carnosol activate AMPK, suppress expressions of gluconeogenic and lipogenic genes, and inhibit proliferation of HepG2 cells. Int. J. Mol. Sci. 22, 4040. 10.3390/ijms22084040 33919842 PMC8070802

[B14] Heim P. Morandi C. Brouwer G. R. Xu L. Montessuit C. Brink M. (2020). Neuregulin-1 triggers GLUT4 translocation and enhances glucose uptake independently of insulin receptor substrate and ErbB3 in neonatal rat cardiomyocytes. Biochim. Biophys. Acta. Mol. Cell Res. 1867, 118562. 10.1016/j.bbamcr.2019.118562 31669265

[B15] Honkoop H. de Bakker D. E. Aharonov A. Kruse F. Shakked A. Nguyen P. D. (2019). Single-cell analysis uncovers that metabolic reprogramming by ErbB2 signaling is essential for cardiomyocyte proliferation in the regenerating heart. Elife 8, e50163. 10.7554/eLife.50163 31868166 PMC7000220

[B16] Kang Y. E. Kim J. M. Choung S. Joung K. H. Lee J. H. Kim H. J. (2016). Comparison of serum Neuregulin 4 (Nrg4) levels in adults with newly diagnosed type 2 diabetes mellitus and controls without diabetes. Diabetes Res. Clin. Pract. 117, 1–3. 10.1016/j.diabres.2016.04.007 27329015

[B17] Kimura H. Nagoshi T. Oi Y. Yoshii A. Tanaka Y. Takahashi H. (2021). Treatment with atrial natriuretic peptide induces adipose tissue browning and exerts thermogenic actions *in vivo* . Sci. Rep. 11 (1), 17466. 10.1038/s41598-021-96970-9 34465848 PMC8408225

[B18] Kocak M. Z. Aktas G. Atak B. M. Duman T. T. Yis O. M. Erkus E. (2020). Is Neuregulin-4 a predictive marker of microvascular complications in type 2 diabetes mellitus? Eur. J. Clin. Invest. 50, e13206. 10.1111/eci.13206 31999832

[B19] Kocak M. Z. Aktas G. Erkus E. Yis O. M. Duman T. T. Atak B. M. (2019). Neuregulin-4 is associated with plasma glucose and increased risk of type 2 diabetes mellitus. Swiss Med. Wkly. 149, w20139. 10.4414/smw.2019.20139 31656034

[B20] Kurek Eken M. Sahin Ersoy G. Yayla Abide C. Sanverdi İ. Devranoglu B. Kutlu T. (2019). Association between circulating neuregulin 4 levels and metabolic, aterogenic, and AMH profile of polycystic ovary syndrome. J. Obstet. Gynaecol. 39, 975–980. 10.1080/01443615.2019.1581754 31064233

[B21] Kurek Eken M. Yayla Abide C. Sahin Ersoy G. Altun Ensari T. Pekin O. Cevik O. (2018). Clinical significance of neuregulin 4 (NRG4) in gestational diabetes mellitus. Gynecol. Endocrinol. 34, 605–608. 10.1080/09513590.2017.1420772 29282998

[B22] López-Soldado I. Niisuke K. Veiga C. Adrover A. Manzano A. Martínez-Redondo V. (2016). Neuregulin improves response to glucose tolerance test in control and diabetic rats. Am. J. Physiol. Endocrinol. Metab. 310, E440–E451. 10.1152/ajpendo.00226.2015 26714846

[B23] Meyer D. Yamaal T. Garratt A. Riethmacher-Sonnenberg E. Kane D. Theill L. E. (1997). Isoform-specific expression and function of neuregulin. Development 124, 3575–3586. 10.1242/dev.124.18.3575 9342050

[B24] Ortega F. J. Mercader J. M. Moreno-Navarrete J. M. Nonell L. Puigdecanet E. Rodriquez-Hermosa J. I. (2015). Surgery-induced weight loss is associated with the downregulation of genes targeted by MicroRNAs in adipose tissue. J. Clin. Endocrinol. Metab. 100, E1467–E1476. 10.1210/jc.2015-2357 26252355

[B25] Patel T. P. Rawal K. Soni S. Gupta S. (2016). Swertiamarin ameliorates oleic acid induced lipid accumulation and oxidative stress by attenuating gluconeogenesis and lipogenesis in hepatic steatosis. Biomed. Pharmacother. 83, 785–791. 10.1016/j.biopha.2016.07.028 27490779

[B26] Rosell M. Kaforou M. Frontini A. Okolo A. Chan Y. W. Nikolopoulou E. (2014). Brown and white adipose tissues: Intrinsic differences in gene expression and response to cold exposure in mice. Am. J. Physiol. Endocrinol. Metab. 306, E945–E964. 10.1152/ajpendo.00473.2013 24549398 PMC3989735

[B27] Suárez E. Bach D. Cadefau J. Palacín M. Zorzano A. Gumà A. (2001). A novel role of neuregulin in skeletal muscle: Neuregulin stimulates glucose uptake, glucose transporter translocation, and transporter expression in muscle cells. J. Biol. Chem. 276, 18257–18264. 10.1074/jbc.M008100200 11278386

[B28] Wang G. X. Zhao X. Y. Meng Z. X. Kern M. Dietrich A. Chen Z. (2014). The brown fat-enriched secreted factor Nrg4 preserves metabolic homeostasis through attenuation of hepatic lipogenesis. Nat. Med. 20, 1436–1443. 10.1038/nm.3713 25401691 PMC4257907

[B29] Wang R. Yang F. Qing L. Huang R. Liu Q. Li X. (2019). Decreased serum neuregulin 4 levels associated with non-alcoholic fatty liver disease in children with obesity. Clin. Obes. 9, e12289. 10.1111/cob.12289 30411515

[B30] Wang S. Jung S. Ko K. S. (2022). Effects of amino acids supplementation on lipid and glucose metabolism in HepG2 cells. Nutrients 14, 3050. 10.3390/nu14153050 35893906 PMC9332103

[B31] Wang T-Y. Liu C. Wang A. Sun Q. (2015). Intermittent cold exposure improves glucose homeostasis associated with brown and white adipose tissues in mice. Life Sci. 139, 153–159. 10.1016/j.lfs.2015.07.030 26281919 PMC4598301

[B32] Wang W. Zhang Y. Yang C. Wang Y. Shen J. Shi M. (2019). Feature Article: Transplantation of neuregulin 4-overexpressing adipose-derived mesenchymal stem cells ameliorates insulin resistance by attenuating hepatic steatosis. Exp. Biol. Med. 244, 565–578. 10.1177/1535370219839643 PMC6545697 30935234

[B33] Wang Y. Huang S. Yu P. (2019). Association between circulating neuregulin4 levels and diabetes mellitus: A meta-analysis of observational studies. PLoS One 14, e0225705. 10.1371/journal.pone.0225705 31815951 PMC6901220

[B34] Yang F. Zhou N. Zhu X. Min C. Zhou W. Li X. (2021). n-3 PUFAs protect against adiposity and fatty liver by promoting browning in postnatally overfed male rats: a role for NRG4. J. Nutr. Biochem. 93, 108628. 10.1016/j.jnutbio.2021.108628 33705952

[B35] Zeng F. Wang Y. Kloepfer L. A. Wang S. Harris R. C. (2018). ErbB4 deletion predisposes to development of metabolic syndrome in mice. Am. J. Physiol. Endocrinol. Metab. 315, E583–E593. 10.1152/ajpendo.00166.2018 29944391 PMC6230712

[B36] Zhang P. Kuang H. He Y. Idiga S. O. Li S. Chen Z. (2018). NRG1-Fc improves metabolic health via dual hepatic and central action. JCI insight 3, 98522. 10.1172/jci.insight.98522 29515030 PMC5922292

[B37] Zhu B. Mei W. Jiao T. Yang S. Xu X. Yu H. (2020). Neuregulin 4 alleviates hepatic steatosis via activating AMPK/mTOR-mediated autophagy in aged mice fed a high fat diet. Eur. J. Pharmacol. 884, 173350. 10.1016/j.ejphar.2020.173350 32726654

